# Supplementary material for: Expression of Aquaglyceroporins in Spermatozoa from Wild Ruminants Is Influenced by Photoperiod and Thyroxine Concentrations
Source: Int J Mol Sci. 2022 Mar 8;23(6):2903. doi: 10.3390/ijms23062903 (PMC8950870; doi:10.3390/ijms23062903)
Supplement: Supplementary file 1 [file ijms-23-02903-s001.zip › ijms-1612040-supplementary.pdf]

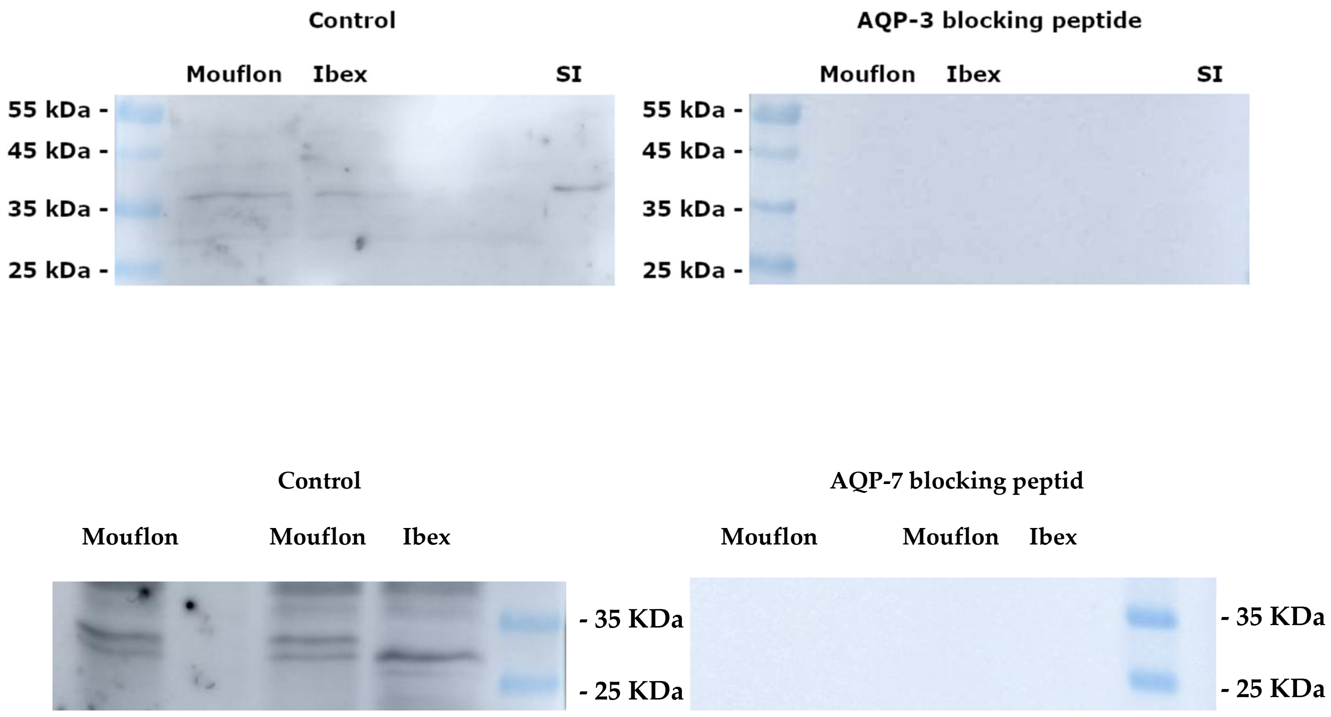


**Supplementary Figure S1:** Western blot images showing expression patterns of AQP3 (above) and AQP7 (below) in positive control (sperm samples of mouflon and ibex containing 35 x $10^6$ spermatozoa, and small intestine tissue lysate (left) and blots resulting from incubations with the AQP3-blocking peptide and AQP7- blocking peptide (right). AQP10-blocking peptide is not available by Abcam, and thus the specificity of the AQP10 antibody was not assessed by peptide competition assay.

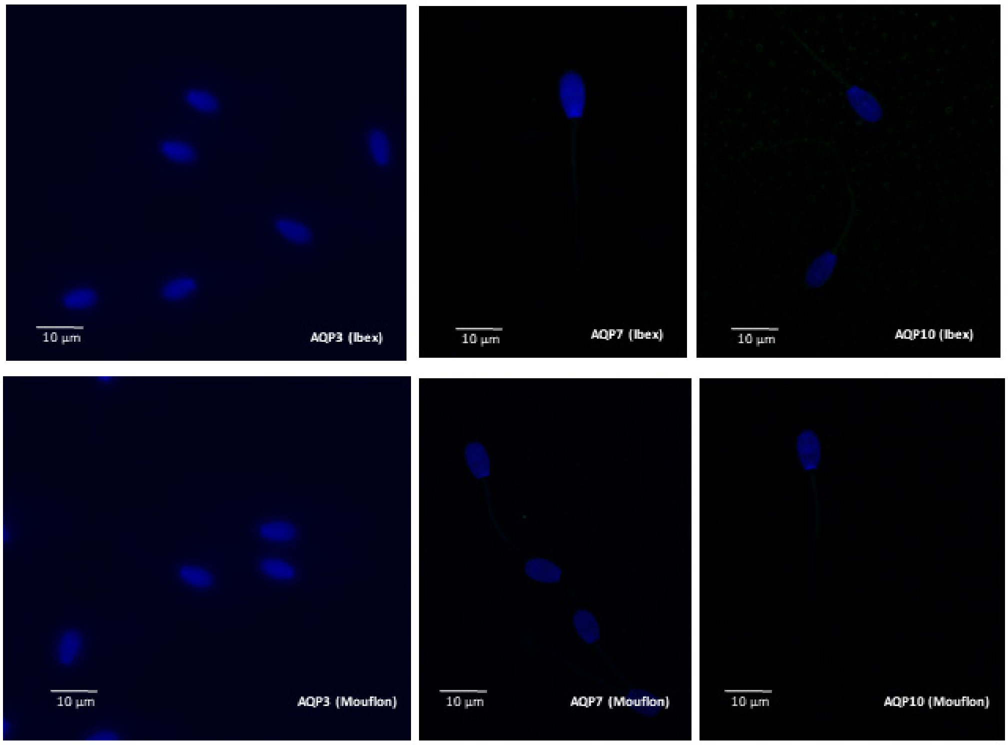


**Supplementary Figure S2:** Negative controls (sample incubated only with secondary antibody, omitting the primary antibody step) for immunolabeling of AQP3, AQP7, and AQP10 in ibex and mouflon sperm.
